# Supplementary material for: Lack of ITS sequence homogenization in Erysimum species (Brassicaceae) with different ploidy levels
Source: Sci Rep. 2022 Oct 7;12:16907. doi: 10.1038/s41598-022-20194-8 (PMC9546898; doi:10.1038/s41598-022-20194-8)
Supplement: Supplementary file 1 — Supplementary Information. [file 41598_2022_20194_MOESM1_ESM.pdf]

Supplementary Material

Figure S1. Boxplot depicting the nucleotide diversities (π) of ITS1 and ITS2 for *Erysimum mediohispanicum*.

Figure S2. Correlation between ploidy level and nucleotide diversity for ITS1.

Table S1. The number of sequences after quality trimming and after cd-hit clustering for all the samples.

Table S2. Nucleotide and haplotype diversity for *E. baeticum* ITS1 and ITS2, at the three-level analyzed.

Table S3. Nucleotide and haplotype diversity for *E. bastetanum* ITS1 and ITS2, at the three-level analyzed.

Table S4. Nucleotide and haplotype diversity for *E. fitzii* ITS1 and ITS2, at the two-level analyzed.

Table S5. Nucleotide and haplotype diversity for *E. lagascae* ITS1 and ITS2, at the three-level analyzed.

Table S6. Nucleotide and haplotype diversity for *E. mediohispanicum* ITS1 and ITS2, at the three-level analyzed.

Table S7. Nucleotide and haplotype diversity for *E. nevadense* ITS1 and ITS2, at the three-level analyzed.

Table S8. Nucleotide and haplotype diversity for *E. popovi* ITS1 and ITS2, at the three-level analyzed.

Table S9. Number of total haplotypes, frequency of each haplotype (based on the total of sequences after cd-hit analysis), number of haplotypes shared among different populations from the same species, and number of haplotypes shared among *E. baeticum* and other *Erysimum* species studied here.

Table S10. Number of total haplotypes, frequency of each haplotype (based on the total of sequences after cd-hit analysis), number of haplotypes shared among different populations from the same species, and number of haplotypes shared among *E. bastetanum* and other *Erysimum* species studied here.

Table S11. Number of total haplotypes, frequency of each haplotype (based on the total of sequences after cd-hit analysis), and number of haplotypes shared among *E. fitzii* and other *Erysimum* species studied here.

Table S12. Number of total haplotypes, frequency of each haplotype (based on the total of sequences after cd-hit analysis), and number of haplotypes shared among *E. lagascae* and other *Erysimum* species studied here.

Table S13. Number of total haplotypes, frequency of each haplotype (based on the total of sequences after cd-hit analysis), number of haplotypes shared among different populations from the same species, and number of haplotypes shared among *E. mediohispanicum* and other *Erysimum* species studied here.

Table S14. Number of total haplotypes, frequency of each haplotype (based on the total of sequences after cd-hit analysis), number of haplotypes shared among different populations from the same species, and number of haplotypes shared among *E. nevadense* and other *Erysimum* species studied here.

Table S15. Number of total haplotypes, frequency of each haplotype (based on the total of sequences after cd-hit analysis), number of haplotypes shared among different populations from the same species, and number of haplotypes shared among *E. popovii* and other *Erysimum* species studied here.

Figure S1. Boxplot depicting the nucleotide diversities (π) of ITS1 and ITS2 for *Erysimum mediohispanicum*. Populations Em21 and Em39 were diploids, and Em71 was polyploid (4×).

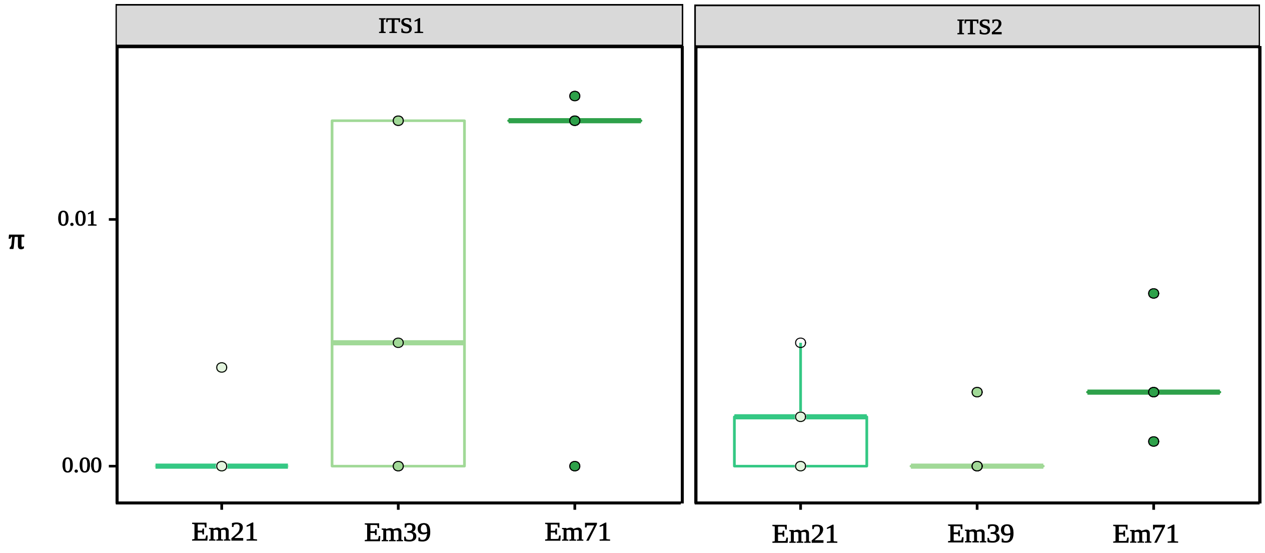

Figure S2. Correlation between ploidy level and nucleotide diversity for ITS1 samples (Spearman's rho: 0.48, p-value: 2.10 x 10^{-6}). The ploidy level for the samples was: 2× (diploid), 4× (tetraploid), 8× (octoploid), and 10× (decaploid).

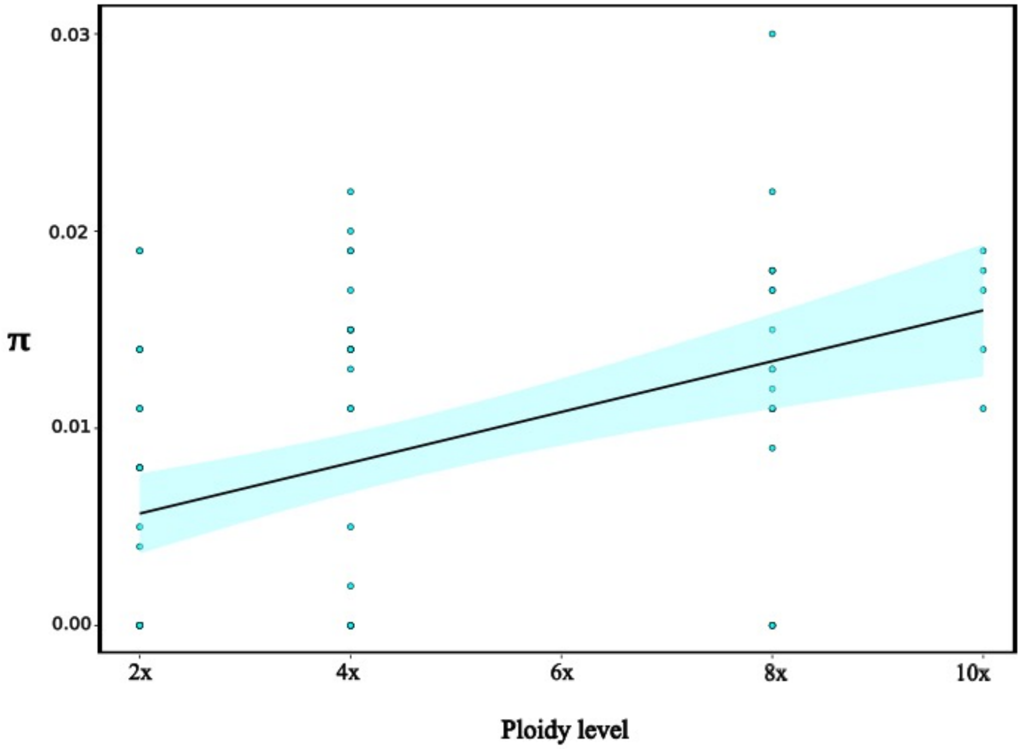

Table S1. Number of sequences after quality trimming and after cd-hit clustering for all the samples.

Taxon	Sample	Number of sequences after quality trimming		Number of sequences after clustering	
		ITS1	ITS2	ITS1	ITS2
E. baeticum	Ebb07-1	9,323	194,745	7,122	191,686
	Ebb07-2	7,358	25,941	6,539	25,311
	Ebb07-3	5,852	45,680	5,274	18,641
	Ebb07-4	5,198	334,388	3,731	132,263
	Ebb07-5	6,092	403,632	5,063	128,485
	Ebb10-1	7,090	608,256	5,289	247,990
	Ebb10-2	9,843	181,762	9,171	65,007
	Ebb10-3	6,710	181,726	6,155	109,565
	Ebb10-4	9,468	352,662	8,618	152,423
	Ebb10-5	10,847	381,902	9,190	160,503
	Ebb12-1	9,820	452,562	8,586	172,469
	Ebb12-2	6,487	600,370	5,328	262,497
	Ebb12-3	6,706	441,938	5,727	167,879
	Ebb12-4	6,013	493,274	5,403	201,674
	Ebb12-5	8,379	71,674	6,784	29,745
E. bastetanum	Ebt01-1	6,673	147,372	5,469	140,547
	Ebt01-2	4,309	19,851	3,581	18,981
	Ebt01-3	6,389	136,348	5,869	133,498
	Ebt01-4	10,349	10,913	9,876	10,627
	Ebt01-5	2,407	25,871	1,916	25,497
	Ebt12-1	9,387	74,828	8,459	73,287
	Ebt12-2	3,417	77,159	2,575	70,854
	Ebt12-3	6,613	31,839	4,897	30,580
	Ebt12-4	7,133	0	5,651	0
	Ebt12-5	4,648	0	4,306	0
	Ebt13-1	5,007	37	4,460	52
	Ebt13-2	6,116	127	5,061	95
	Ebt13-3	6,075	518	5,443	356
	Ebt13-4	4,898	180	4,279	90
	Ebt13-5	7,209	412	6,616	311
E. fitzii	Ef01-1	2,459	1,144	504	666
	Ef01-2	36,284	3,208	27,977	2,992
	Ef01-3	20,575	12,272	18,112	11,221
	Ef01-4	21,007	3,040	19,382	2,819
	Ef01-5	24,480	0	21,220	0
E. lagascae	Ela07-1	46,652	7,949	38,755	7,498
	Ela07-2	28,675	25,037	26,098	23,325
	Ela07-3	43,375	44,502	43,522	40,688
	Ela07-4	37,367	26,642	34,303	23,085
	Ela07-5	21,787	68,778	18,544	49,696
E. mediohispanicum	Em21-1	1,057	46	1,027	38
	Em21-2	1,517	23	1,470	17
	Em21-3	1,500	37	1,454	34
	Em21-4	1,061	54	1,013	47
	Em21-5	2,460	31	1,801	27

	Em39-1	740	10	580	7
	Em39-2	3,860	36	355	36
	Em39-3	4,184	48	297	39
	Em39-4	6,317	61	401	59
	Em39-5	1,542	12	156	11
	Em71-1	3,372	324	310	259
	Em71-2	3,152	92	281	68
	Em71-3	5,526	253	418	187
	Em71-4	1,817	260	215	165
	Em71-5	3,824	52	387	23
E. nevadense	En05-1	19,550	3,660	22,540	3,260
	En05-2	33,125	3,361	29,703	3,222
	En05-3	20,691	38,519	19,623	36,417
	En05-4	72,945	9,749	65,114	8,588
	En05-5	101	6,507	72	5,839
	En10-1	17,010	3,031	15,179	2,734
	En10-2	15,650	70,323	13,255	65,241
	En10-3	21,251	62,475	17,700	60,745
	En10-4	32,198	66,797	30,531	63,176
	En10-5	19,828	182,495	17,403	175,659
	En12-1	14,249	101,910	13,363	100,098
	En12-2	15,973	100,528	13,990	97,509
	En12-3	18,005	6,829	16,465	6,298
	En12-5	29,274	291,336	26,163	287,162
E. popovii	Ep16-1	10,691	32,788	8,965	27,961
	Ep16-2	2,753	26,133	2,590	15,418
	Ep16-3	5,647	26,132	5,300	25,074
	Ep16-4	6,081	18,519	4,967	17,651
	Ep16-5	6,922	14,860	6,381	13,983
	Ep20-1	19,099	55,464	13,841	48,638
	Ep20-2	11,313	15,011	10,034	13,546
	Ep20-3	4,570	7,092	4,108	5,309
	Ep20-4	5,056	6,522	3,753	3,234
	Ep20-5	5,861	25,346	5,119	22,381
	Ep27-1	7,621	58,789	5,962	53,469
	Ep27-2	6,239	44,639	5,227	36,287
	Ep27-3	15,831	12,787	14,370	11,745
	Ep27-4	6,207	38,248	5,147	35,449
	Ep27-5	25,310	24,543	21,224	236

Table S2. Nucleotide and haplotype diversity for *E. baeticum* ITS1 and ITS2, at the three-level (species, population, individuals) analyzed.

E. baeticum	**Sample code**	**ITS1**		**ITS2**	
		**π**	**Hd**	**π**	**Hd**
**Species level**	Ebb	0.013	0.983	0.006	0.897
**Population level**	Ebb07	0.015	1.000	0.009	0.933
	Ebb10	0.012	0.963	0.004	1.000
	Ebb12	0.015	1.000	0.008	0.933
**Individual level**	Ebb07-1	0.022	1.000	0	0
	Ebb07-2	0.011	1.000	0	0
	Ebb07-3	0	0	0.010	1.00
	Ebb07-4	0.012	1.000	0	0
	Ebb07-5	0.018	1.000	0	0
	Ebb10-1	0.018	1.000	0	0
	Ebb10-2	0	0	0	0
	Ebb10-3	0.011	1.000	0	0
	Ebb10-4	0	0	0	0
	Ebb10-5	0.017	1.000	0	0
	Ebb12-1	0.009	1.000	0	0
	Ebb12-2	0.011	1.000	0.008	1.000
	Ebb12-3	0	0	0	0
	Ebb12-4	0.015	1.000	0	0
	Ebb12-5	0.011	1.000	0	0

Table S3. Nucleotide and haplotype diversity for *E. bastetanum,* ITS1 and ITS2 samples, at the three-level analyzed.

E. bastetanum	**Sample code**	**ITS1**		**ITS2**	
		**π**	**Hd**	**π**	**Hd**
**Species level**	Ebt	0.013	0.983	0.006	0.893
**Population level**	Ebt01	0.013	0.969	0.005	0.694
	Ebt12	0.012	0.991	0.008	0.900
	Ebt13	0.013	0.975	0.005	0.916
**Individual level**	Ebt01-1	0.013	1.000	0.005	0.694
	Ebt01-2	0.014	1.000	0	0
	Ebt01-3	0.020	1.000	0.008	1.000
	Ebt01-4	0.019	1.000	0.021	1.000
	Ebt01-5	0.017	1.000	0.005	0.666
	Ebt12-1	0.015	1.000	0.010	1.000
	Ebt12-2	0.015	1.000	0	0
	Ebt12-3	0.015	1.000	0	0
	Ebt12-4	0.013	1.000	0	0
	Ebt12-5	0.011	1.000	0	0
	Ebt13-1	0.013	1.000	0.004	1.000
	Ebt13-2	0.018	1.000	0.004	1.000
	Ebt13-3	0.017	1.000	0.006	1.000
	Ebt13-4	0.013	1.000	0.005	1.000
	Ebt13-5	0.030	1.000	0.006	1.000

Table S4. Nucleotide and haplotype diversity for *E. fitzii,* ITS1 and ITS2 samples, at the two level analyzed.

E. fitzii	**Sample code**	**ITS1**		**ITS2**	
		**π**	**Hd**	**π**	**Hd**
**Species level**	Ef	0.011	0.944	0.009	0.972
**Individual level**	Ef01-1	0	0	0.012	1.000
	Ef01-2	0	0	0.010	1.000
	Ef01-3	0.008	1.000	0.008	1.000
	Ef01-4	0	0	0.010	1.000
	Ef01-5	0.019	1.000	0	0

Table S5. Nucleotide and haplotype diversity for *E. lagascae,* ITS1 and ITS2 samples, at the three-level analyzed.

E. lagascae	**Sample code**	**ITS1**		**ITS2**	
		**π**	**Hd**	**π**	**Hd**
**Species level**	Ela	0.011	1.00	0.005	0.733
**Individual level**	Ela07-1	0	0	0	0
	Ela07-2	0.008	1.00	0	0
	Ela07-3	0	0	0	0
	Ela07-4	0	0	0.007	1.000
	Ela07-5	0.019	1.00	0	0

Table S6. Nucleotide and haplotype diversity for *E. mediohispanicum,* ITS1 and ITS2 samples, at the three-level analyzed.

E. mediohispanicum	Sample code	ITS1		ITS2	
		π	Hd	π	Hd
**Species level**	Em	0.300	0.969	0.003	0.805
**Population level**	Em21	0.001	0.400	0.002	0.750
	Em39	0.001	0.955	0.001	0.333
	Em71	0.003	0.963	0.003	0.847
**Individual level**	Em21-1	0.004	0.812	0.005	1.000
	Em21-2	0	0	0.002	1.000
	Em21-3	0	0	0.002	1.000
	Em21-4	0	0	0	0
	Em21-5	0	0	0	0
	Em39-1	0.014	1.000	0.003	1.000
	Em39-2	0	0	0	0
	Em39-3	0	0	0	0
	Em39-4	0.005	1.000	0	0
	Em39-5	0.014	1.000	0	0
	Em71-1	0.014	1.000	0.007	1.000
	Em71-2	0.014	1.000	0.003	1.000
	Em71-3	0	0	0.003	1.000
	Em71-4	0.014	1.000	0.001	1.000
	Em71-5	0.015	1.000	0.003	1.000

Table S7. Nucleotide and haplotype diversity for *E. nevadense,* ITS1 and ITS2 samples, at the three-level analyzed.

E. nevadense	Sample code	ITS1		ITS2	
		π	Hd	π	Hd
**Species level**	En	0.010	0.938	0.006	0.941
**Population level**	En05	0.008	0.785	0.010	0.933
	En10	0.004	0.800	0.007	1.000
	En12	0.009	0.952	0.003	0.833
**Individual level**	En05-1	0.008	1.000	0.007	1.000
	En05-2	0	0	0	0
	En05-3	0	0	0	0
	En05-4	0.008	1.000	0	0
	En05-5	0.011	1.000	0	0
	En10-1	0	0	0	0
	En10-2	0	0	0	0
	En10-3	0	0	0	0
	En10-4	0	0	0	0
	En10-5	0.008	1.000	0	0
	En12-1	0	0	0	0
	En12-2	0.014	1.000	0	0
	En12-3	0.011	1.000	0	0
	En12-4	0	0	0	0
	En12-5	0	0	0	0

Table S8. Nucleotide and haplotype diversity for E. popovii, ITS1 and ITS2 samples, at the three-level analyzed.

E. popovii	Sample code	ITS1		ITS2	
		π	Hd	π	Hd
**Species level**	Ep	0.015	0.984	0.007	0.943
**Population level**	Ep16	0.013	0.977	0.007	0.977
	Ep20	0.016	1.000	0.008	0.944
	Ep27	0.004	0.888	0.004	0.866
**Individual level**	Ep16-1	0	0	0.010	1.000
	Ep16-2	0.014	1.000	0.011	1.000
	Ep16-3	0.011	1.000	0	0
	Ep16-4	0.019	1.000	0.005	1.000
	Ep16-5	0.022	1.000	0.008	1.000
	Ep20-1	0.019	1.000	0.010	1.000
	Ep20-2	0.017	1.000	0.010	1.000
	Ep20-3	0.014	1.000	0.005	1.000
	Ep20-4	0.011	1.000	0.010	1.000
	Ep20-5	0.018	1.000	0	0
	Ep27-1	0	0	0	0
	Ep27-2	0	0	0	0
	Ep27-3	0.005	1.000	0	0
	Ep27-4	0	0	0.008	1.000
	Ep27-5	0.002	1.000	0	0

Table S9. Number of total haplotypes, frequency of each haplotype as relative abundance (based on the total of sequences after cd-hit analysis), number of haplotypes shared among different populations from the same species, and number of haplotypes shared among *E. baeticum* and the other *Erysimum* species studied here.

E. baeticum		**ITS1**				**ITS2**			
	**Sample**	**Number**	**Relative abundance (%)**	**Shared with other populations**	**Shared with other species**	**Number**	**Relative abundance (%)**	**Shared with other populations**	**Shared with other species**
**Species level**	Ebb	31	H1: 10.25<br>H2: 7.69<br>H3-H5: 5.12<br>H6-H31: 2.56	3	0	9	H1: 29.41<br>H2-H5: 11.76<br>H6-H9: 5.88	5	0
**Population level**	Ebb07	13	H1-H13: 7.69	H1: Ebb12		4	H1-H2: 33.33<br>H3-H4: 16.66	H1: Ebb12<br>H2: Ebb10, Ebb12	
	Ebb10	9	H1: 33.33<br>H2-H9: 8.33	H1: Ebb12		4	H1: 40<br>H2-H4: 20	H2: Ebb07, Ebb12<br>H3: Ebb12<br>H6: Ebb12	
	Ebb12	11	H1-H3: 14.28<br>H4-H11: 7.14	H2: Ebb07<br>H3: Ebb10		4	H1: 50<br>H2-H4: 16.66	H1: Ebb07<br>H2: Ebb12, Ebb07<br>H3: Ebb10<br>H4: Ebb10	
**Individual level**	Ebb07-1	2	H1: 64.55<br>H2: 11.84			1	H1: 98.42		
	Ebb07-2	3	H1: 51.38<br>H2: 24.58<br>H3: 12.89			2	H1: 64.67<br>H2: 32.90		
	Ebb07-3	1	H1: 90.12			1	H1: 40.41		
	Ebb07-4	3	H1: 31.89<br>H2: 27.79<br>H3: 12.08			1	H1: 39.55		
	Ebb07-5	4	H1: 54.89<br>H2: 16.00<br>H3: 6.18<br>H4: 6.02			1	H1: 31.83		
	Ebb10-1	5	H1: 31.01<br>H2: 18.22<br>H3: 10.23<br>H4: 8.29<br>H5: 6.82			1	H1: 40.77		
	Ebb10-2	1	H1: 93.17			1	H1: 35.76		
	Ebb10-3	2	H1: 80.78<br>H2: 10.93			1	H1: 60.29		
	Ebb10-4	1	H1: 91.02			1	H1: 43.22		
	Ebb10-5	3	H1: 61.98<br>H2: 17.56<br>H3: 5.18			1	H1: 40.02		
	Ebb12-1	3	H1: 55.44<br>H2: 19.68<br>H3: 12.30			2	H1: 22.50<br>H2: 15.69		
	Ebb12-2	3	H1: 61.30<br>H2: 11.94<br>H3: 8.87			1	H1: 43.72		
	Ebb12-3	1	H1: 85.40			1	H1: 37.98		
	Ebb12-4	5	H1: 38.78<br>H2: 15.61<br>H3: 15.20<br>H4: 12.68<br>H5: 7.56			1	H1: 40.88		
	Ebb12-5	2	H1: 42.54<br>H2: 38.41			1	H1: 41.50		

Table S10. Number of total haplotypes, frequency of each haplotype (based on the total of sequences after cd-hit analysis), number of haplotypes shared among different populations from the same species, and number of haplotypes shared among *E. bastetanum* and other *Erysimum* species studied here.

E. bastetanum		**ITS1**				**ITS2**			
	**Sample**	**Number**	**Relative abundance (%)**	**Shared with other populations**	**Shared with other species**	**Number**	**Relative abundance (%)**	**Shared with other populations**	**Shared with other species**
**Species level**	Ebt	38	H1: 10.86<br>H2: 6.52<br>H3: 4.34<br>H4-H38: 2.17	2	0	17	H1: 27.5<br>H2: 15<br>H3-H4: 10<br>H5-H6: 5<br>H7-H17: 2.5	2	0
**Population level**	Ebt01	9	H1: 25<br>H2: 16.6<br>H3-H9: 8.33	H1:Ebt12, Ebt13<br>H2: Ebt13		4	H1: 55.55<br>H2: 22.22<br>H3-H4: 11.11	H1: Ebt12	
	Ebt12	15	H1: 12.5<br>H2: 6.25<br>H3-H15: 6.25	H1: Ebt01, Ebt13		4	H1: 40<br>H2-H4: 20	H1: Ebt01<br>H2: Ebt13	
	Ebt13	13	H1: 5.55<br>H2-H13: 5.55	H1: Ebt01, Ebt12<br>H2: Ebt01		4	H1: 60.25<br>H2: 17.32<br>H3: 15.94<br>H4: 3.82	H2: Ebt12	
**Individual level**	Ebt01-1	3	H1: 43.75<br>H2: 28.68<br>H3: 9.5			1	H1: 95.36		
	Ebt01-2	2	H1: 59.43<br>H2: 23.67			1	H1: 95.61		
	Ebt01-3	2	H1: 61.91<br>H2: 29.94			2	H1: 83.54<br>H2: 14.36		
	Ebt01-4	2	H1: 74.73<br>H2: 20.69			2	H1: 91.87<br>H2: 5.51		
	Ebt01-5	3	H1: 46.94<br>H2: 22.18<br>H3: 10.46			2	H1: 86.81<br>H2: 11.74		
	Ebt12-1	3	H1: 75.14<br>H2: 9.05<br>H3: 5.91			3	H1: 59.53<br>H2: 31.10<br>H3: 7.31		
	Ebt12-2	3	H1: 41.94<br>H2: 21.94<br>H3: 11.91			1	H1: 91.82		
	Ebt12-3	4	H1: 33.87<br>H2: 17.93<br>H3: 11.81<br>H4: 10.43			1	H1: 96.04		
	Ebt12-4	4	H1: 33.38<br>H2: 25.34<br>H3: 15.49<br>H4: 5			0	0		
	Ebt12-5	2	H1: 84.18<br>H2: 8.45			0	0		
	Ebt13-1	3	H1: 55.78<br>H2: 23.92<br>H3: 9.36			5	H1: 30<br>H2: 27.27<br>H3: 21.21<br>H4: 15.15<br>H5: 6		
	Ebt13-2	5	H1: 38.84<br>H2: 20.40<br>H3: 9.66<br>H4: 7.66<br>H5: 6.16			4	H1: 46<br>H2: 22.10<br>H3: 18.94<br>H4: 12.63		
	Ebt13-3	2	H1: 53.444<br>H2: 36.14			3	H1: 57.02<br>H2: 26.40<br>H3: 16.57		
	Ebt13-4	3	H1: 34.01<br>H2: 27.41<br>H3: 25.92			3	H1: 63.33<br>H2: 24.44<br>H3: 12.22		
	Ebt13-5	5	H1: 37.57<br>H2: 31.77			3	H1: 72.34<br>H2: 14.14		

H3: 10.75<br>H4: 5.85<br>H5: 5.81	H3: 9.6

Table S11. Number of total haplotypes, frequency of each haplotype (based on the total of sequences after cd-hit analysis), and number of haplotypes shared among *E. fitzii* and other *Erysimum* species studied here.

E. fitzii		ITS1			ITS2		
	Sample	Number	Relative abundance (%)	Shared with other species	Number	Relative abundance (%)	Shared with other species
**Species level**	Ef01	6	H1-H3: 22.22<br>H4-H6: 11.11	0	7	H1-H7: 14.27	0
**Individual level**	Ef01-1	1	H1: 20		1	H1: 83.07	
	Ef01-2	4	H1: 27.42<br>H2: 24.04<br>H3: 20.34<br>H4: 5.27		1	H1: 91.01	
	Ef01-3	1	H1: 88.02		2	H1: 51.63<br>H2: 42.97	
	Ef01-4	1	H1: 92.26		2	H1: 81.30<br>H2: 10.50	
	Ef01-5	2	H1: 58.84<br>H2: 27.83		1	H1: 85.16	

Table S12. Number of total haplotypes, frequency of each haplotype (based on the total of sequences after cd-hit analysis), and number of haplotypes shared among *E. lagascae* and other *Erysimum* species studied here.

E. lagascae		ITS1			ITS2		
	**Sample**	**Number**	**Relative abundance (%)**	**Shared with other species**	**Number**	**Relative abundance (%)**	**Shared with other species**
**Species level**	Ela07	7	H1-H7: 14.27	0	2	H1: 74.98<br>H2: 19.34	0
**Individual level**	Ela07-1	1	H1: 83.07		1	H1: 93.16	
	Ela07-2	1	H1: 91.01		1	H1: 91.42	
	Ela07-3	2	H1: 57.63<br>H2: 42.97		1	H1: 97.55	
	Ela07-4	2	H1: 81.30<br>H2: 10.50		2	H1: 46.94<br>H2: 25.31	
	Ela07-5	1	H1: 85.16		1	H1: 94.5	

Table S13. Number of total haplotypes, frequency of each haplotype as relative abundance (based on the total of sequences after cd-hit analysis), number of haplotypes shared among different populations from the same species, and number of haplotypes shared among *E. mediohispanicum* and the other *Erysimum* species studied here.

E. mediohispanicum		**ITS1**				**ITS2**			
	**Sample**	**Number**	**Relative abundance (%)**	**Shared with other populations**	**Shared with other species**	**Number**	**Relative abundance (%)**	**Shared with other populations**	**Shared with other species**
**Species level**	Em	10	H1: 23.67<br>H2: 22.79<br>H3: 20.25<br>H4: 12.54<br>H5: 9.92<br>H6:2.92<br>H7: 2.41<br>H8-H10: 1.5	1	5	13	H1: 37.70<br>H2: 18.80<br>H3: 16.07<br>H4: 10.19<br>H5: 4<br>H6: 4.14<br>H7: 1.98<br>H8: 1.73<br>H9-H13: 1	0	0
**Population level**	Em21	2	H1: 63.79<br>H2: 36.20			4	H1: 44.85<br>H2: 36.02<br>H3: 11.74<br>H4: 5.14		
	Em39	3	H1: 19<br>H2: 14<br>H3: 14	H1: Em71	H1: Ebt12, Ebt13, Ebt01, En12<br>H5: En10	1	H1: 96.64		
	Em71	5	H1-H3: 10<br>H4-H5: 7	H1: Em39	H1: Ebt12, Ebt13, Ebt01, En12<br>H2: Ebt01, Ebt12, Ebt13<br>H3: Ebt13<br>H4: Ebt13	5	H1: 25.92<br>H2: 19.65<br>H3: 18.66<br>H4: 17.52<br>H5: 7.12		
**Individual level**	Em21-1	1	H1: 97.16			4	H1: 39.47<br>H2: 31.57<br>H3: 21.05<br>H4: 7.89		
	Em21-2	1	H1: 96.90			2	H1: 70.58<br>H2: 29.41		
	Em21-3	1	H1: 96.93			3	H1: 58.82<br>H2: 32.35<br>H3: 8.82		
	Em21-4	2	H1: 65.97<br>H2: 29.50			4	H1: 63.82<br>H2: 19.14<br>H3: 8.51<br>H4: 8.51		
	Em21-5	1	H1: 73.21			1	H1: 100		
	Em39-1	3	H1: 51.03<br>H2: 39.31<br>H3: 9.65			2	H1: 55.55<br>H2: 44.44		
	Em39-2	5	H1: 33.34<br>H2: 28.14<br>H3: 17.51<br>H4: 12.45<br>H5: 8.51			2	H1: 57.14<br>H2: 42.85		
	Em39-3	2	H1: 85.52<br>H2: 14.47			8	H1: 80.55<br>H2-H8: 2.77		
	Em39-4	1	H1: 100			1	H1: 100		
	Em39-5	4	H1: 48.44<br>H2: 25.77<br>H3: 16.14<br>H4: 9.62			1	H1: 100		
	Em71-1	4	H1: 52.22<br>H2: 36.75<br>H3: 5.55<br>H4: 5.54			1	H1: 100		
	Em71-2	4	H1: 48.06<br>H2: 24.11<br>H3: 18.49<br>H4: 9.32			4	H1: 38.61<br>H2: 31.27<br>H3: 18.91<br>H4: 11.19		
	Em71-3	3	H1: 46.24<br>H2: 39.33			3	H1: 52.94<br>H2: 29.41		

		H3: 13.77		H3: 17.64
Em71-4	2	H1: 74<br>H2: 26	5	H1: 25.66<br>H2: 23.52<br>H3: 20.32<br>H4: 17.64<br>H5: 12.83
Em71-5	3	H1: 44.27<br>H2: 41.42<br>H3: 14.30	4	H1: 49.69<br>H2: 23.63<br>H3: 13.93<br>H4: 12.72

Table S14. Number of total haplotypes, frequency of each haplotype as relative abundance (based on the total of sequences after cd-hit analysis), number of haplotypes shared among different populations from the same species, and number of haplotypes shared among *E. nevadense* and the other *Erysimum* species studied here.

E. nevadense		ITS1				ITS2			
	**Sample**	**Number**	**Relative abundance (%)**	**Shared with other populations**	**Shared with other species**	**Number**	**Relative abundance (%)**	**Shared with other populations**	**Shared with other species**
**Species level**	En	15	H1: 23,80<br>H2: 14.28<br>H3-H15: 4.7	4	H1: Ebt. Em<br>H47: Ebt<br>H66: Ef<br>H81: Em	12	H1-H2: 16.66<br>H3-H4: 11.11<br>H5-H12: 5.55	4	0
**Population level**	En05	5	H1: 50<br>H2: 12.5<br>H3: 12.5<br>H4: 12.5<br>H5: 12.5	H1,H2: En10<br>H3: En12	H1: Ebt12<br>H2: Ef<br>H4: En12, Em71, Em39, Ebt13, Ebt12, Ebt01	6	H1: 28.57<br>H2-H6: 14.28	H1: En10<br>H2: En12<br>H4: En12	
	En10	4	H1: 50<br>H2: 16.66<br>H3: 16.66<br>H4: 16.66	H1,H2: En05<br>H4: En12	H3: Em39	7	H1-H7: 14.28	H1: En05<br>H3: En12	
	En12	7	H1-H7: 14.28	H4: En10<br>H3: En05	H4: En05. Em71. Em39. Ebt13. Ebt12. Ebt01	3	H1: 50<br>H2-H3: 25	H2: En05<br>H4: En05	
**Individual level**	En05-1	1	H1: 86.73			2	H1: 77.20<br>H2: 11.88		
	En05-2	2	H1: 76.73<br>H2: 12.93			1	H1: 91.81		
	En05-3	1	H1: 93.09			1	H1: 94.54		
	En05-4	2	H1: 59.24<br>H2: 30.02			1	H1: 95.78		
	En05-5	2	H1: 58.41<br>H2: 12.87			1	H1: 89.73		
	En10-1	1	H1: 89.23			2	H1: 78.78<br>H2: 11.41		
	En10-2	1	H1: 84.69			1	H1: 92.77		
	En10-3	1	H1: 83.29			1	H1: 97.23		
	En10-4	1	H1: 94.82			1	H1: 94.57		
	En10-5	2	H1: 53.33<br>H2: 34.43			2	H1: 61.68<br>H2: 34.57		
	En12-1	1	H1: 93.78			1	H1: 98.22		
	En12-2	2	H1: 76.02<br>H2: 11.55			1	H1: 96.99		
	En12-3	3	H1: 39.48<br>H2: 26.18<br>H3: 25.77			1	H1: 92.22		
	En12-4	-							
	En12-5	1	H1: 89.37			1	H1: 98.56		

Table S15. Number of total haplotypes, frequency of each haplotype (based on the total of sequences after cd-hit analysis), number of haplotypes shared among different populations from the same species, and number of haplotypes shared among *E. popovii* and other *Erysimum* species studied here.

E. popovii		ITS1				ITS2			
	Sample	Number	Relative abundance (%)	Shared with other populations	Shared with other species	Number	Relative abundance (%)	Shared with other populations	Shared with other species
**Species level**	Ep	30	H1: 8.5<br>H2: 8.5<br>H3: 5.7<br>H4-H30: 2.85	1	0	19	H1: 18.51<br>H2: 14.81<br>H3-H19: 3.70	1	0
**Population level**	Ep16	12	H1-H12: 8.33			9	H1: 20<br>H2-H9: 10	H1: Ep20, Ep27	
	Ep20	12	H1: 15.38<br>H2-H12: 7.69	H1: Ep27		9	H1-H2: 18.18<br>H3-H9: 9	H1: Ep27, Ep16	
	Ep27	7	H1: 30<br>H2: 20<br>H3-H7: 10	H1: Ep20		4	H1-H2: 33.33<br>H3-H4: 16.66	H1: Ep16, Ep20	
**Individual level**	Ep16-1	2	H1: 65.55<br>H2: 18.30			4	H1: 42.33<br>H2: 18.43<br>H3: 16.87<br>H4: 7.64		
	Ep16-2	2	H1: 61.09<br>H2: 32.98			3	H1: 25.23<br>H2: 21.94<br>H3: 11.82		
	Ep16-3	2	H1: 62.93<br>H2: 30.91			1	H1: 95.95		
	Ep16-4	2	H1: 75.46<br>H2: 6.21			2	H1: 85.98<br>H2: 9.33		
	Ep16-5	4	H1: 32.72<br>H2: 27.70<br>H3: 26.19<br>H4: 5.56			2	H1: 87.94<br>H2: 6.15		
	Ep20-1	2	H1: 44.87<br>H2: 27.59			2	H1: 78.89<br>H2: 8.79		
	Ep20-2	3	H1: 72.67<br>H2: 8.49<br>H3: 7.52			2	H1: 78.82<br>H2: 11.41		
	Ep20-3	2	H1: 80.70<br>H2: 9.19			2	H1: 67.72<br>H2: 7.13		
	Ep20-4	3	H1: 48.23<br>H2: 20.90<br>H3: 5.08			2	H1: 61.34<br>H2: 28.57		
	Ep20-5	3	H1: 61.28<br>H2: 14.43<br>H3: 11.61			1	H1: 88.30		
	Ep27-1	1	H1: 78.23			1	H1: 90.95		
	Ep27-2	2	H1: 78.36<br>H2: 5.41			2	H1: 41.02<br>H2: 40.26		
	Ep27-3	3	H1: 49.91<br>H2: 27.51<br>H3: 13.34			2	H1: 67.41<br>H2: 24.43		
	Ep27-4	2	H1: 75.88<br>H2: 7.04			2	H1: 84.08<br>H2: 8.59		
	Ep27-5	2	H1: 69.96<br>H2: 13.89			2	H1: 90.20<br>H2: 6.19		
